# Nurses’ and community support workers’ experience of telehealth: a longitudinal case study

**DOI:** 10.1186/1472-6963-14-164

**Published:** 2014-04-10

**Authors:** Urvashi Sharma, Malcolm Clarke

**Affiliations:** 1 Department of Computer Science, Brunel University, Kingston Lane, Uxbridge, Middlesex, UK

**Keywords:** Experience of threat, Telehealth, Nurses, Community support workers, Interpretative phenomenological analysis (IPA)

## Abstract

**Background:**

Introduction of telehealth into the healthcare setting has been recognised as a service that might be experienced as disruptive. This paper explores how this disruption is experienced.

**Methods:**

In a longitudinal qualitative study, we conducted focus group discussions prior to and semi structured interviews post introduction of a telehealth service in Nottingham, U.K. with the community matrons, congestive heart failure nurses, chronic obstructive pulmonary disease nurses and community support workers that would be involved in order to elicit their preconceptions and reactions to the implementation.

**Results:**

Users experienced disruption due to the implementation of telehealth as threatening. Three main factors add to the experience of threat and affect the decision to use the technology: change in clinical routines and increased workload; change in interactions with patients and fundamentals of face-to-face nursing work; and change in skills required with marginalisation of clinical expertise.

**Conclusion:**

Since the introduction of telehealth can be experienced as threatening, managers and service providers should aim at minimising the disruption caused by taking the above factors on board. This can be achieved by employing simple yet effective measures such as: providing timely, appropriate and context specific training; provision of adequate technical support; and procedures that allow a balance between the use of telehealth and personal visit by nurses delivering care to their patients.

## Background

In this paper, we explore how changes necessitated by the introduction of telehealth are experienced by those charged with delivering telehealth care on the ground. Furthermore, we consider whether these changes are seen to constitute a threat and, where applicable, to identify the domains within which the threat is experienced.

Telehealth has many definitions, and can be broadly understood as remote patient monitoring consisting of two distinct yet integrated parts. First is the technological modality that involves collecting physiological data from patients and sending it through telephone, fax, e-mail or videoconferencing. The second part is the care delivery process, where the technological intervention is complemented by nurse case management or medical support through call centre assistance. The main aim of telehealth services is to improve clinical outcomes alongside a more efficient use of clinician time [1-3]. In this study, the telehealth service enables collection of remote patient data such as blood glucose level, weight, blood pressure, oxygen level and heart rate. This physiological data is sent using a standard telephone connection to a remote server. Received data is then studied by an elected clinical team member responsible for ensuring that any immediate alerts are addressed.

Despite government backed initiatives and research finding indicating improved healthcare outcomes, telehealth has not been widely integrated alongside standard care in the United Kingdom (U.K.) [4-10]. It is clear from the growing literature that the use of technology such as telehealth is a function of a complex interplay of technological as well as social and organisational issues, where it is suggested that the success of technology in healthcare is dependent on the “match” of these factors [4-9,11-16]. For example, problems and even the failure of the telehealth initiative may result from discrepancies between the perspectives of healthcare stakeholders, the aspirations of the administrators and the capabilities and limitations of the healthcare technology [4-9,13-17]. The extant literature also acknowledges that the change in an organisation occasioned by the introduction of new technology causes its users to feel threatened [18,19].

It is, therefore, important to understand how key clinical users make sense of the introduction of telehealth service, especially of those who are more intimately involved with the everyday telehealth routines such as nurses and technical staff [1,2,20]. In particular, ascertaining how the challenges of integrating telehealth are experienced as threatening and the effect of such experiences on telehealth technology use will enhance understanding user behaviour towards technology initiated change, especially in the field of remote patient monitoring. Thus, with the aim of facilitating a nuanced exploration of key aspects of the social and organisational context that affects the implementation and use of telehealth and to understand the experiences of clinical users over time, this study, adopted a longitudinal qualitative approach. In addition, to capture the tacit and ephemeral nature of aspects that emerge in medical practice during daily interactions, this research adopted a phenomenological method of inquiry, imposing no theoretical underpinning and assumption on the findings. Instead, it emphasises the users’ experience within a given context. The focus of this research is purely upon how the users describe their experience of the challenges posed by integrating telehealth with existing case management practice and care routines.

## Methods

### Implementation site

Nottingham is the seventh largest urban area in the U.K. and has a population of around 950,000 [21]. It has a large older population suffering from one or more chronic conditions. The Telehealth project began in 2007 as a pilot. With initial study results showing reduced hospital admission and increase in capacity of case managers, in 2009, The Nottingham City Primary Care Trust (PCT) decided to implement telehealth on a larger scale alongside case management provided by community matrons (CM), chronic heart failure (CHF) nurses and chronic obstructive pulmonary disease (COPD) nurses. The PCT also recruited community support workers (CSWs) to assist nurses with equipment installation and data management. The study aimed at recruiting a minimum of 250 patients to whom telehealth equipment was to be allocated.

### Participants

A purposive sample of staff participating in this project was recruited after obtaining their consent to take part in the study. Inclusion criteria were that a staff member be one of the four groups of clinical users who were involved with the day-to-day operation of the telehealth service:

1. Community matrons: registered and experienced nurses with knowledge of various Long Term Conditions (LTCs). Their caseload contained patients with complex healthcare needs i.e. those who suffer from two or more LTCs such as asthma, diabetes, COPD and CHF, and who have been admitted to hospital on multiple occasions due to symptom exacerbation.

2. Congestive heart failure nurses: nurses who specialise in CHF. Assigned to CHF patients, their routines include helping patients manage their condition at home and monitor their progress.

3. Chronic obstructive pulmonary disease nurses: nurses who specialise in COPD, and are part of a larger COPD team - an initiative of the COPD INFORCE3 project. They manage patients with severe COPD and monitor their progress.

4. Community support workers: staff recruited to provide technical assistance to nurses and in addition and who carry out the initial assessment of patients for their suitability to receive the telehealth service.

### Data collection

Data was collected through focus group discussions (FGD) prior to introduction of the service and semi-structured interviews post. Both the discussions and interviews were digitally recorded. Participant consent was gained in advance for permission to record and was confirmed in person on the day of the meeting. Ethical approval was granted by the NHS Nottingham ethical committee.

Three focus groups were conducted, with four main groups of clinical users at an early stage of project roll-out in July 2009. Each discussion group consisted of representatives from at least two of the nursing groups. Three focus group discussions were held at different locations in Nottingham and a total of 16 staff members took part, Table 1 elicits the composition of each focus group. Each discussion lasted 40–70 minutes. Staff had experience with telehealth that ranged from 0–6 months. The discussions aimed at eliciting initial perceptions of users and their preconceived notions about telehealth, and specifically if they perceived telehealth as helping or hindering.

**Table 1:** Composition of the focus groups and type of staff interviewed

**Data collection**	**Participants**
Focus group discussions (×3)	
1^st^ Focus group	3 CMs, and 1 CHF nurse
2^nd^ Focus group	1 CHF nurse, 1 COPD nurse and 2 CSWs
3^rd^ Focus group	2 CMs, 2 CHF nurses, 2 COPD nurses and 2 CSWs
Interviews (×8)	2 CMs, 2 CHF nurses, 1 COPD nurse, 1CSW, service manager, and a telehealth/telecare lead

In June 2010, eight in-depth interviews were carried out to explore the actual experiences of the participants with telehealth use, one year after service roll out. Four of the FGD participants also took part in the interviews (see Table 1). There were four new participants, one of whom was the project manager, who was interviewed in order to gain insight on possible expectations of events from a management perspective.

Interviews were held at the participants’ own work place in healthcare centres based in the Nottingham area in order to minimise disruption to the participant’s daily work. Each interview lasted between 45–50 minutes.

### Data analysis

This study adopted interpretative phenomenological analysis (IPA) to collect and analyse data. IPA allows exploring meanings that people ascribe to their lived experiences [22]. It can be argued that three approaches are encompassed within IPA: Husserl’s notion of ‘bracketing’, which requires the researcher to conduct research without any presumptions and focus on descriptions that people provide rather seeking explanations; Heidegger’s notion of ‘hermeneutics’ which asks the researcher to adopt an iterative approach whilst analysing data; and symbolic interactionism which alludes to symbolic meanings that people convey in their interaction [22,23].

Following the IPA methodology, initial reading of each transcript enabled the researcher to become familiar with the description given by each participant of their experience with telehealth. Further reading of the transcripts, allowed themes to emerge. The researcher then started to connect these themes by either clustering or classifying them as superordinate concepts. Resulting themes were tabulated. This process, when repeated for each transcript, allowed the researcher to acknowledge new issues that emerged and identify repeating patterns [23].

## Results

The main theme that emerged from the data analysis was the perception that the telehealth would be a threat to three aspects of their existing roles, namely: (1) daily work routines; (2) interaction with their patients and; (3) skill set and expertise.

### Changes to daily work routine, increased workload and conflicting job roles

The daily work routines and workload of nurses consisted of a range of complex processes and practices. In this context, the key theme that emerged was that telehealth service perturbed users’ routines and added to their workload because of equipment installation and maintenance, use of computer system to access data, patient assessment and education. In addition, the service also caused confusion over newly assigned job roles.

Nurses felt that having to install telemonitoring equipment in the patients’ home was an unacceptable imposition on their existing workloads, and said that it was not part of their role. Unnecessary workload demands came from duplicate processes such as the need to enter the same patient information in two data systems, a community matron explains how it impacted her: “*that is very very time consuming, when you are having to actually document something twice. You are doing on telehealth and having to transfer it to SystemOne”* (FGD 2009).

However, after a year, the responsibility of installing the equipment was delegated to engineers. In addition, the nurses felt that although the telehealth increased workload and affected work practices, there were positive outcomes, such as expressed by a COPD nurse: “*because if somebody is unwell, you know, traditionally a patient may well sit there at home, feeling unwell. But they would not necessarily call us. They would leave it a few days until they get worse, and then call us. However, with the monitoring, we are able to, because their oxygen level is low on that day, we would call them. So it may have increased the workload, but in a good way, because it might mean that we visit them earlier and, but we are, you know, we are in the business of preventing admissions to hospital. So the workload is a correct way of our time, it’s the correct use, that’s fine, absolutely fine*” COPD (Interview 2010).

However, the situation with data entry (use of two systems) did not change and it was foreseen that this might “*continue for a long time*” (manager) and it was noted this had been an on-going issue for almost 4 years and, with lack of funding, change seemed even less likely.

Introduction of CSWs resulted in another change in the daily routine of the nurses. Although the nurses were involved in the training of the CSWs, they were unclear of the role of the CSWs, and what could be expected of the CSWs. Indeed, discussions during the focus groups were used to clarify their exact remit. Confusion was also felt by CSWs as they resisted against the expectations that CSWs should be active in equipment installation: “*it’s not what I thought I took the job for. I thought it was personalised, clinical and computer and documenting*” (sighs) CSW (FGD 2009).

In subsequent interviews, the CSWs reported that their role in the telehealth service had become more clearly defined and that they had gained more confidence and established a good reputation. Nurses agreed with this and argued that what had originally been felt as a cause of disruption to routine had now become a valuable resource: “*We couldn’t do telehealth without her (CSW), to be honest……She’s taken it on. She’s very competent anyway, but she’s taken it on to check the results every day, and then just lets us know of any alerts. And it’s really noticeable when she’s off. It’s something that we don’t have to think about to go and do, because it’s not in our normal routine*” CHF1 (Interview 2010).

With respect to patient training, the nurses and CSWs remained responsible for educating the patients on the use of the telehealth equipment and when asked if they would prefer an engineer to deliver the initial training on use of telehealth equipment insisted on retaining this responsibility because: “*I think it’s easier to explain something to somebody when you’ve got some sort of relationship with them, whereas an engineer comes in who hasn’t got the relationship, could be over-technical because I know engineers who are very over-technical and leave them more confused than when they walked through the door, whereas we know the patients, we know who’d need in-depth information and we know the ones that need to keep it simple and, you know, we can give them the information they want*” CSW (Interview 2010).

### Changes to interaction with patients

Nurses considered telehealth as a ‘monitoring tool’ and expressed concern should the telemonitoring technology become a substitute for human presence. They questioned the effectiveness of the tool to diagnose underlying health problems and argued that it could contribute to social exclusion.

Participants were initially apprehensive about the decreased face-to-face interaction with patients that might result from using the telehealth service and how this might hinder accurate assessment and diagnosis of a patient’s condition. They argued that subjective information from the appearance of a patient can be vital in diagnosing underlying conditions such as depression and infection: “*I think the sort of thing one wonders with is lack of the face-to-face contact. And although you are asking questions and doing specific…you know…sign and symptoms there is always a chance that there could be something that you are only going to see if you are face-to-face with somebody*” CHF nurse (FGD 2009).

Some nurses said that telehealth could contribute to the social exclusion of patients as many patients were old and lived alone with no immediate social contact nearby: “*if you get them to the clinic or a group session or we were visiting, we could identify social isolation. And perhaps day centres, things like that. So it can mask I think social isolation. This patient was crying out that I just wanted the human touch back. He was stir crazy and he could not get out of the house and felt he was confined to the house. Just talking about things to someone and not to a box makes difference… because it’s (telehealth equipment) not a person, is it?*” CHF nurse (FGD 2009).

In the subsequent interviews, nurses still held the strong feeling that telehealth was not an alternative to face-to-face visits. However, they described telehealth as a ‘monitoring tool’ which helped them to be more reactive to exacerbation events, make informed decisions regarding patient treatment and communicate relevant information effectively to their patients.

The nurses also described how they had become aware of where telehealth intervention was useful and where the equipment had its limitations: “*lot of patients with cardiac disease have arrhythmia so their heart rates are very irregular and we know that some of the actual equipment doesn’t pick up an accurate heart rate so it’s always good to check yourself and, you know, be able to be comfortable with that and it doesn’t pick up irregular heart rhythms. It wouldn’t tell you that suddenly that patient’s gone from a regular heart rhythm to an irregular and it’s very common in heart failure for someone to be suddenly having a normal heart rate and then them to go into arrhythmia which could be quite life threatening so I think from that point of view I would still need to physically assess my patients*” CHF2 (Interview 2010).

### Changes to skill set and marginalisation of expertise

Nurses felt that their expertise was being undermined and challenged by the telehealth system. This was exacerbated by what was considered to be inappropriate training and the lack of technical support.

According to the staff, the training provided was incomplete, untimely and with no follow-up. In some cases, training was missed completely, leaving the users confused and frustrated. As recounted by a community matron (FGD 2009): *“the computer had gone down so we did not actually have any training on how to access the site. So we muddled through with it”.*

Moreover, participants felt that the training did not recognise the realities of nursing work or the context of the work setting. Nurses emphasised that if the technology is to measure physiological parameters of patient then a volunteer should have been used during the training session rather than a plastic dummy. They argued that using a plastic dummy masked many of the real life scenarios where taking readings becomes challenging.

A further aspect of the lack of support was the promise of assistance from community support workers to deal with technological issues. However, due to delays in recruitment, nurses had to resolve many of the technological issues themselves which challenged their confidence: *“I am not technological. I am not familiar with the setup of it. I am happy to check clinical parameters and assess the patient but the technical side of it is not just my bag really and I would not feel confident to say that yes I have set this up and it’s working and its safe… and nothing wrong is gonna happen”* Community Matron (FGD 2009).

With time, issues of training and support were addressed. The project manager acknowledged the impact of untimely training: *“In hindsight we would not have trained so many staff all at once, because people got the training, we, sort of, did large numbers very early on. Bang, bang, bang, got everybody trained up, but they didn’t all go out and start using it straightaway. So, by the time they came to use it, they’d lost the skills”* (Interview 2010).

One clinical user, whilst reflecting on the support arrangements of having a qualified engineer for equipment installation and its maintenance at the patient home, wondered why such provisions were not made any sooner: “*We had a meeting last week to introduce us to the girl who’s going to be running it, and we were told that there were two engineers that were going to do a day each devoted to telehealth. So, it’s only just happening now…after a year!*” CHF1 (interview 2010).

## Discussion

**Figure 1:**
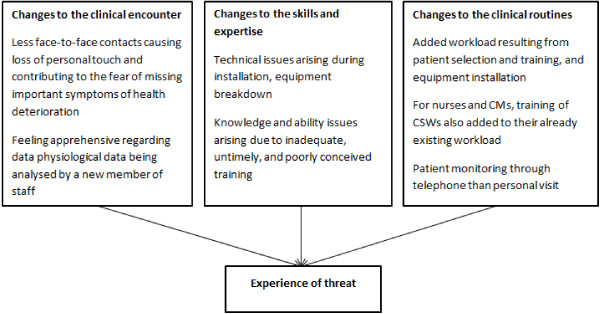
Diagrammatical representation of the themes.

Integration of telehealth into mainstream care can be difficult. Exploring what hinders and what helps integration from the staff perspective can help managers and policy makers to appreciate the complex dynamics. Furthermore, with recent studies questioning the cost-effectiveness of telehealth [24], it becomes even more salient to unravel such underlying issues. In this study, three focus group discussions were carried out at the beginning of the project followed by eight in-depth interviews a year later, to explore how telehealth is perceived and later experienced by nurses and community support workers. Data analysis using IPA revealed three high level themes (as depicted in Figure 1) which aimed at describing the experience of threat by participants as a result of telehealth implementation.

The first theme revealed how changes to daily work were experienced as threatening. Research suggests that practitioners establish daily routines in order to cope with the chaotic, tacit and ephemeral nature of medical work; and by introducing changes, for example by introducing a telehealth service, routines are transformed [25,26]. In our study, participants, in particular, the community matrons and nurses mainly talked about changes to their daily work routines in terms of workload and work processes. Nurses felt that the introduction of telehealth caused a marked increase in their workload and claimed this was located around the installation of equipment, and initial patient assessment. The reason for the change in work processes was due to the change in delivery of the care process. Where before nurses had to visit most of the patients in their caseload every day, now they had to contact their patients by telephone after looking at their daily observations. Such threat was not experienced by the CSWs as they were newly recruited and were adjusting to their new role, specified around the integrated service.

The second theme emerged from participants’ description of experiencing threat due to telehealth changing interaction with patients. Community matrons and nurses explained how subjective data (the appearance of the patient) was as valuable as objective data (e.g. blood pressure, weight). An encounter between patient and nurse is termed a ‘social encounter’; where the healthcare professional interacts with the patient to develop a relationship that includes understanding the patient not just as a biomedical subject with non-functional bodily aspects, but as an individual with a social and personal background and needs [27-30]. On several occasions, matrons and nurses mentioned that telehealth changed their encounter with patients. Regular personal visits were to be cut, and instead, be replaced by telephone calls discussing patients’ physiological symptoms. This instilled a sense of worry among some staff members as they feared missing vital health symptoms, such as onset of an infection. This was combined with an apprehensive altitude resulting from delegating the task of analysing patient data to CSWs. Others on the other hand, recounted providing assurance to the patient on the continuity of personal visits as they reported some patients lacking any other form of social contact.

The third theme emerged from the participants’ reflection on how telehealth challenged their technical expertise and skill set. CMs, nurses, and CSWs perceived this as a threat to their professional calibre, and at times, strongly condemned provisions of introducing telehealth service. Indeed, the telehealth was found challenging to use [31], but there were many comments on the lack of adequate training and support. Such provision is acknowledged within literature as ‘facilitating conditions’ [32], and is often cited as affecting decision by users to use the technology [1,2].

The longitudinal nature of this study (over 12 months) allowed user experience over time to be elicited, and capture the shift in users understanding of how telehealth could be employed. However, although the clinical users understood the telehealth and its capabilities, they still could not justify its use in this context. This was in part due to the nurses being used to travelling and visiting patients at home and were reluctant to see this aspect change. The only role that telehealth was seen to play was to allow prioritisation of visits based on daily observations of the patients and in some cases, where a visit was not warranted, cancel the visit.

### Implications of this study

This study by exploring the experiences of nurses and technical staff on the use of telehealth identifies how changes in routines, interactions, and expertise of the user may be necessary. It highlights that such changes carry a sense of threat and are experienced as threatening by the users, affecting the overall telehealth service use. By focusing our work on the more immediate impact of a change (experienced as a threat at a personal and professional level), the study reveals the salience of timely mediation of such a threat. This may be vital, as the resistance could result in a complete disregard of the technology. In addition, the study also recognised that users perceive telehealth as a tool that aids in monitoring and not as a complete substitution for face-to-face interactions.

### Limitations of this study

This study has several limitations. The findings are based on a single-study design that is based upon the views of selected set of clinical users in one telehealth service in a specific area. Therefore caution is required to generalise the results from this study to either another country or different clinical user population. In the pre-implementation phase of the service and due to the demanding schedule of nursing duties, only a few participants were available to take part in the focus groups. The study investigates a service that is based on an integrated model of telehealth and case management to deliver healthcare to patients with chronic disease. Other telehealth services may be based on a different model of healthcare delivery.

## Conclusion

This study determines that telehealth providers and managers who are responsible for procuring and implementing services need to take note of user experience, and especially of non-technical users such as nurses and community support workers. The study highlights that introducing and implementing a telehealth service that is to be integrated into main stream bring many changes to the clinical routines of the user, interaction with patients and expertise of the user, all of which can be experienced as threatening. If adequate steps are not taken and the concerns of users are not addressed in a timely manner, results can be detrimental to service integration.

## Competing interests

The authors declare that they have no competing interests.

## Authors’ contributions

US conducted research, analysed data, and drafted the manuscript. MC conceived of the study, its design and helped in drafting the manuscript. Both authors read and approved the final manuscript.

## Pre-publication history

The pre-publication history for this paper can be accessed here:

http://www.biomedcentral.com/1472-6963/14/164/prepub
